# Single-track sequencing for genotyping of multiple SNPs in the N-acetyltransferase 1 (NAT1) gene

**DOI:** 10.1186/1472-6750-4-28

**Published:** 2004-11-25

**Authors:** Pavel Soucek, Camilla Furu Skjelbred, Marit Svendsen, Tom Kristensen, Elin H Kure, Vessela N Kristensen

**Affiliations:** 1 Group for Biotransformations, Center of Occupational Diseases, National Institute of Public Health, Praha 10, Czech Republic; 2 Department of Environmental and Health Studies, Faculty of Arts and Sciences, Telemark University College, Norway; 3 Department of Occupational and Environmental Medicine, Telemark Central Hospital, 3710 Skien, Norway; 4 Department of Biochemistry, University of Oslo; 5 Department of Pathology, Ullevål University Hospital, Oslo, Norway; 6 Department of Genetics, Norwegian Radium Hospital, Montebello 0310, Oslo, Norway; 7 Advanced Technology Center National Cancer Institute, NIH, NCI, Bethesda

## Abstract

**Background:**

Fast, cheap and reliable methods are needed to identify large populations, which may be at risk in relation to environmental exposure. Polymorphisms in NAT1 (N-acetyl transferase) may be suitable markers to identify individuals at risk.

**Results:**

A strategy allowing to address simultaneously 24 various genetic variants in the NAT1 gene using the single sequencing reaction method on the same PCR product is described. A modified automated DNA sequencing using only one of the sequence terminators was used to genotype PCR products in single-track sequencing reactions of NAT1 and was shown to be universal for both DNA sequencing using labeled primers and labeled nucleotides. By this method we detected known SNPs at site T640G, which confers the NAT1*11 allele with frequency of 0.036, further T1088A and C1095A with frequency of 0.172 and 0.188, respectively and a deletion of TAATAATAA in the poly A signal area with a frequency 0.031. All observed frequencies were in Hardy Weinberg equilibrium and comparable to those in Caucasian population. The single-track signatures of the variant genotypes were verified on samples previously genotyped by RLFP.

**Conclusions:**

The method could be of great help to scientists in the field of molecular epidemiology of screening of large populations for known informative biomarkers of susceptibility, such as NAT1.

## Background

We have previously described the single sequencing reaction (SSR) protocol for assessment of a known polymorphism in the 3'utr region of *CYP19 *(aromatase) [1,2]. Here we have extended and optimized the use of the method for multiple polymorphisms in the *NAT1 *gene. The increasing number of detected mutations in the *NAT1*, makes genotyping using conventional restriction fragment length polymorphism (RFLP) or allele-specific amplification complicated. We developed a rapid and universal strategy based on single-track DNA sequencing analysis of a unique PCR product encompassing the entire *NAT1 *coding region (contains no introns) along with the flanking 5' and 3' untranslated regions. It allows a rapid and economic characterization of *NAT1 *alleles. Our method brings reproducible results on both Alf Express™ (Pharmacia) and ABI310 PRISM sequencing instruments and may be adopted for majority of epidemiological studies with relevance of *NAT1 *in environmentally related diseases.

N-acetyltransferases (NAT, EC 2.3.1.5) are implicated in the biotransformation of primary arylamines (e.g. 2-naphthylamine and aminobiphenyls), heterocyclic amines, hydrazines, and their N-hydroxylated metabolites present in tobacco smoke and food [3-7]. An increased activity for p-aminobenzoic acid acetylation (marker for NAT1 activity) was observed in the breast malignant tissues compared to benign and control tissues [8,9]. Human NATs may have adapted a common catalytic mechanism from cysteine proteases for acetyl-transfer reactions [10,11]. The *NAT1 *gene is highly polymorphic (for listing of known variant alleles ). Expression of NAT1*16 but not NAT1*10 and NAT1*11 caused a 2-fold decrease in the amount and catalytic activity of NAT1 in COS-1 cell cytosol [12-14]. All available data suggest that slow NAT1 phenotype results from *NAT1 *allelic variants that encode reduced expression of *NAT1 *and/or less-stable NAT1 protein [15].

Epidemiological studies suggest that the *NAT1 *and *NAT2 *acetylation polymorphisms modify the risk of developing urinary bladder, colorectal, breast, head and neck, lung, and possibly prostate cancers [16-18]. Interactions between NAT2*4 and NAT1*10 were suggested by the increased frequency of the NAT2*4/NAT1*10 haplotype [19-21]. The individual risks associated with NAT1 acetylator phenotypes/genotypes are usually small, but they increase when considered in conjunction with other susceptibility genes and/or aromatic and heterocyclic amine carcinogen exposures. Because of the relatively high frequency of the variant *NAT1 *genotypes in the population, the attributable cancer risk may be high. Large-scale molecular epidemiological studies that investigate the role of *NAT1 *genotypes and/or phenotypes together with other genetic susceptibility gene polymorphisms and biomarkers of carcinogen exposure are necessary to expand our current understanding of the role of NAT1 acetylation polymorphisms in cancer risk [22].

## Results

**Figure 1:**
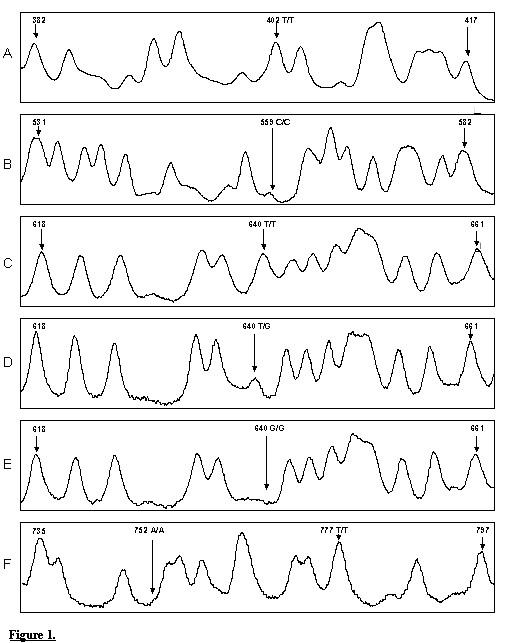
Genotyping of the coding region of *NAT1 *by T-track sequencing on Alf Express™. (A) Displayed area from T382 to T417 (B) Displayed area from T531 to T582 (C) Displayed area from T618 to T661, with genotypes *640T/T*; (D) *640T/G*; (E) *640G/G*; (F) – Displayed area from T735 to T797, *A752T *and *T777C *alleles not found.

**Figure 2:**
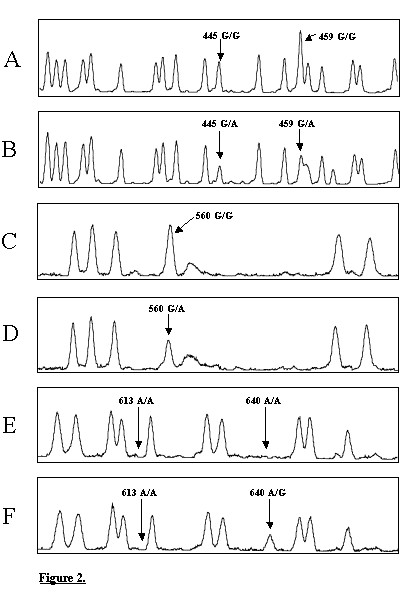
*NAT1 *variants in the coding region assessed by G-track sequencing on ABI310 PRISM. (A) Displayed area from G418 to G476, genotypes *445G/G *and *459G/G*; (B) *445G/A *and *459G/A *heterozygote; (C) Displayed area from G544 to G590, genotypes *560G/G*; (D) *560G/A*; (E) Displayed area from G598 to G655, genotypes *613A/A *and *640T/T*; (F) *613A/A *and *640A/G*.

**Figure 3:**
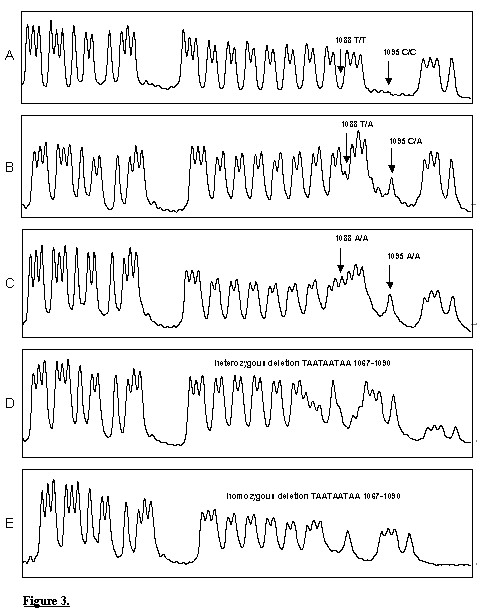
*NAT1 *alleles in 3'-untranslated region assessed by single A-track sequencing on Alf Express™. Displayed area from A1038 to A1104. (A) NAT1*4/*4 – wild type; (B) *1088T/A *and *1095C/A *heterozygote; (C) *1088A/A *and *1095A/A *homozygote; (D) NAT1*4/*11 allele; (E) NAT1*11/*11 allele.

**Figure 4:**
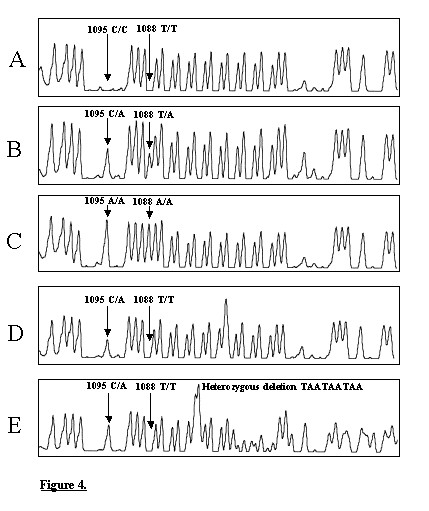
*NAT1 *alleles in 3'-untranslated region by single A-track sequencing on ABI310 PRISM. Displayed area from A1104 to A1048. (A) NAT1*4/*4 – wild type; (B) *1088T/A *and *1095C/A *heterozygote; (C) *1088A/A *and *1095A/A *homozygote; (D) *1088T/T *homozygote and *1095C/A *heterozygote; (E) NAT1*4/*11 allele.

We outlined a strategy allowing to address simultaneously 13 genetic variants in the coding area of *NAT1 *with two sequencing tracks: A and G and 11 variants in the 3' flanking area with two tracks: A and T (Table 1). Using single-track sequencing of the *NAT1 *coding region, we have detected 11 individuals carrying polymorphisms at site *T640G*, which characterizes NAT1*11 alleles (Figure 1C,1D,1E). Identical results were obtained when performing the single-track sequencing on an Alf Express™ system as on ABI310 PRISM (Figure 2E,2F). The frequency of this polymorphism, 0.036 found by single-track sequencing in our study was comparable to the frequency 0.032 found by PCR-RFLP method in 396 German control individuals [23]. Polymorphisms *T1088A *and *C1095A *(Figure 3A,3B,3C and Figure 4A,4B,4C, representing each genotype analyzed by both Alf Express™ and ABI310 PRISM) were in perfect linkage disequilibrium. The frequency of these polymorphisms was 0.172 and 0.188 respectively and was comparable to frequency 0.206 reported by Bruhn *et al*. [24].

A-track sequencing of the 3'-untranslated region of *NAT1 *revealed a deletion of TAATAATAA (Figure 3D,3E on Alf Express™ and Figure 4E on ABI310 PRISM). It was found in 7 individuals accounting for the frequency 0.031, which is comparable to the frequency 0.033 observed in the study on 314 control individuals by Bruhn *et al*. [24]. This change is also unique for the NAT1*11 alleles and co-segregates with *T640G *polymorphism. Very good co-segregation of *T640G *and *1067-1090delTAATAATAA *polymorphisms in 7 informative samples was observed (4 heterozygotes and 3 homozygotes). Despite the different chemistry, Figures 1, 2, 3, 4 demonstrate perfect concordance between the results obtained by Alf Express™ and ABI310 PRISM analysis, verified on samples with previously characterized genotypes by RFLP.

## Discussion

A modified automated DNA sequencing with a fluorescent label was used to genotype PCR products spanning through the whole *NAT1 *gene by single-track sequencing reactions in 192 control individuals. Previously, we have shown that single-track sequencing reactions performed on PCR products, with subsequent analysis on an Alf Express™ DNA Sequencer, can be as informative, sensitive, and accurate as complete sequencing reactions but more economical option for the genotyping of known polymorphisms in the human aromatase gene [1]. Compared to analysis by full gel-based sequencing, four times more samples can be analyzed per gel in considerably shorter time. The method could be particularly efficient in cases of high density of polymorphic sites residing in a common area as in the case of *NAT1*.

## Conclusions

This paper shows that single-track sequencing reactions can be used as a tool for screening for all types of known polymorphisms in field laboratories with limited or overloaded sequence capacity. Compared to standard sequencing, this single track sequencing may have better signal to noise ratio. This approach is time and cost effective and can be accommodated for high-throughput analyses in epidemiology studies.

## Methods

### Materials

Chemicals for PCR and sequencing were purchased from ABI (Applied Biosystems, Foster City, CA, USA) and Amersham Biosciences (Uppsala, Sweden).

### Subjects

The study included 192 DNA samples from Norwegian healthy individuals obtained through Norwegian Population Registry as a population-based series of residents in the Oslo area. Test samples from healthy Norwegian controls with known genotypes (previously assessed by RFLP) were available.

### PCR amplification of NAT1

A single PCR amplification of the entire coding region and the 3' flanking area of *NAT1 *(923 bp) using forward primer: 5'-tactgggctctgaccactat-3' and reverse primer: 5'-tgctttctagcataaatcacc-3' was performed. PCR mix contained 16.9 μl dH_2_O, 10 × PCR buffer (2.5 μl), 1.25 mM MgCl_2 _(4 μl), 2.0 mM dNTP (1.0 μl mixture of each), 10 μM oligonucleotide primer, 0.2 μl Taq DNA polymerase (Perkin Elmer, 5 U/μl) and 1.0 μl of genomic DNA (100 ng/μl) in a final volume of 25 μl. Thermal cycling (GeneAmp 2400, PE, Foster city, CA) included initial denaturation 2 min at 94°C, 10 cycles of 30 sec at 94°C, 30 sec at decreasing annealing temperature 58 to 48°C, 1 min at 72°C, and 25 cycles of 30 sec at 94°C, 30 sec at 50°C and 1 min at 72°C. Quality of PCR products was checked on 6% acrylamide gels.

### Single-track sequencing

The SNPs studied by this assay are summarized in Table 1. Single A-, G- and T- track sequencing using 1 given terminator at a time was performed using both Alf Express™ system (Pharmacia Biotech, Uppsala, Sweden) and ABI310 PRISM capillary sequencer. Alf Express™ Cy5-labeled primers NAT1A-F-CY5: 5'-gggagggtatgtttacagca-3' and NAT1A-POLYA-CY5: 5'-gcataaatcaccaatttcca-3' were used for genotyping the coding region and 3'untranslated area of *NAT1*, respectively. The single sequencing reactions (7 μl) contained: 1.25 μl of dH_2_O, 0.5 μl of 10-times concentrated FS polymerase buffer (125 mM Tris-HCl, pH 9.5, 50 mM (NH_4_)_2_SO_4_, 150 mM MgCl_2_), 2 μl of ddNTP termination mix (containing 10 mM dNTPs and either ddATP or ddTTP (10 μM) for A-track or T-track sequencing respectively, 1 μl of 2 μM primer, 0.25 μl of FS polymerase (5 U/μl), and 2 μl of *NAT1 *PCR product. The following conditions were used for thermal cycling: initial denaturation for 5 min at 95°C, 35 cycles of 30 sec at 95°C, 30 sec at 50°C, and 1 min at 68°C. The single-tracks were evaluated using conventional software AlfWin, Fragment Analyzer, v. 1.02 supplied with the sequencer (Alf Express™). For the single-track sequencing by ABI310 PRISM we used the same sequencing primers as for Alf Express™ system however labeled with the fluorophor 6-FAM. Sequencing reactions (10 μl) contained: 5.0 μl dH_2_O, 1.0 μl of 10-times concentrated Thermo Sequenase buffer (260 mM Tris-HCl, pH 9.5, 65 mM MgCl_2_), 2.0 μl of ddNTP termination mix (containing dNTPs and either ddATP or ddGTP for A-track or G-track sequencing respectively – for composition see Table 3), 0.5 μl of 1 μM primer, 0.5 μl of Thermo Sequenase DNA polymerase (with pyrophosphatase) 3.2 U/μl and 1.0 μl *NAT1 *PCR product (described above). Thermal cycling conditions were as follows: initial denaturation 20 sec at 95°C, 25 cycles of 20 sec at 95°C, 20 sec at 55°C and 1 min at 72°C, and then 10 cycles of 20 sec at 95°C, 1 min at 72°C. Results were evaluated using GeneScan Analysis software, v. 3.7.

## Abbreviations

ABPs – aminobiphenyls, NAT1 – N-acetyltransferase 1, SSR (Single Sequencing Reaction)

## Author's contributions

PS performed the SSR analysis on an Alf Express™ and prepared the first draft of the paper, CFS and MS optimized the SSR analysis for an ABI system in the lab of EHK, TK designed primers and contributed with reagents, materials, and advice, VNK brought the idea, organized the study and was responsible for the revisions of the paper.

## Figures and Tables

**Table 1:** Overview of *NAT1 *alleles and selection for single-track sequencing

**coding region:**	**track/ddNTP**	**allele**
350,351 GG → CC	G	NAT1*5
402 T → C	T	NAT1*20
445 G → A	G	NAT1*11
459 G → A	G	NAT1*11
497–499 GGG → CCC	G	NAT1*5
559 C → T	T	NAT1*15
560 G → A	G	NAT1*14
613 A → G	G	NAT1*21
640 T → G	T/G	NAT1*11
752 A → T	T	NAT1*22
777 T → C	T	NAT1*23, *27
781 G → A	G	NAT1*24
787 A → G	G	NAT1*25
**poly A region:**	**track/ddNTP**	**allele**
884 A → G	A	NAT1*5
976 delA	A	NAT1*5
1025 delT	T	NAT1*29
1067–1090 delTAATAATAA	A	NAT1*11
1067–1090 delTAATAA	A	NAT1*28
1067–1090 delTAA	A	NAT1*18
1067–1090 insTAA	A	NAT1*26
1088 T → A	A	NAT1*10, *14,
		NAT1*18, *29
1091 ins AAA	A	NAT1*16
1095 C → A	A	NAT1*3, *10, *11,
		NAT1*14,*16,*18,
		NAT1*26, *29
1105 delT	T	NAT1*5
